# Supplementary material for: Tead1a Initiates Transcriptional Priming Through the TEAD1a/YAP‐Notch1‐Spi1/Cebpα Axis to Promote Neutrophil Fate
Source: Adv Sci (Weinh). 2025 Aug 28;12(41):e05441. doi: 10.1002/advs.202505441 (PMC12591127; doi:10.1002/advs.202505441)
Supplement: Supplementary file 4 — Supplementary Dataset [file ADVS-12-e05441-s004.zip › 3-2.zip!/3-2/02-2.cluster/cluster3/Enrich/ego_MF.pdf]

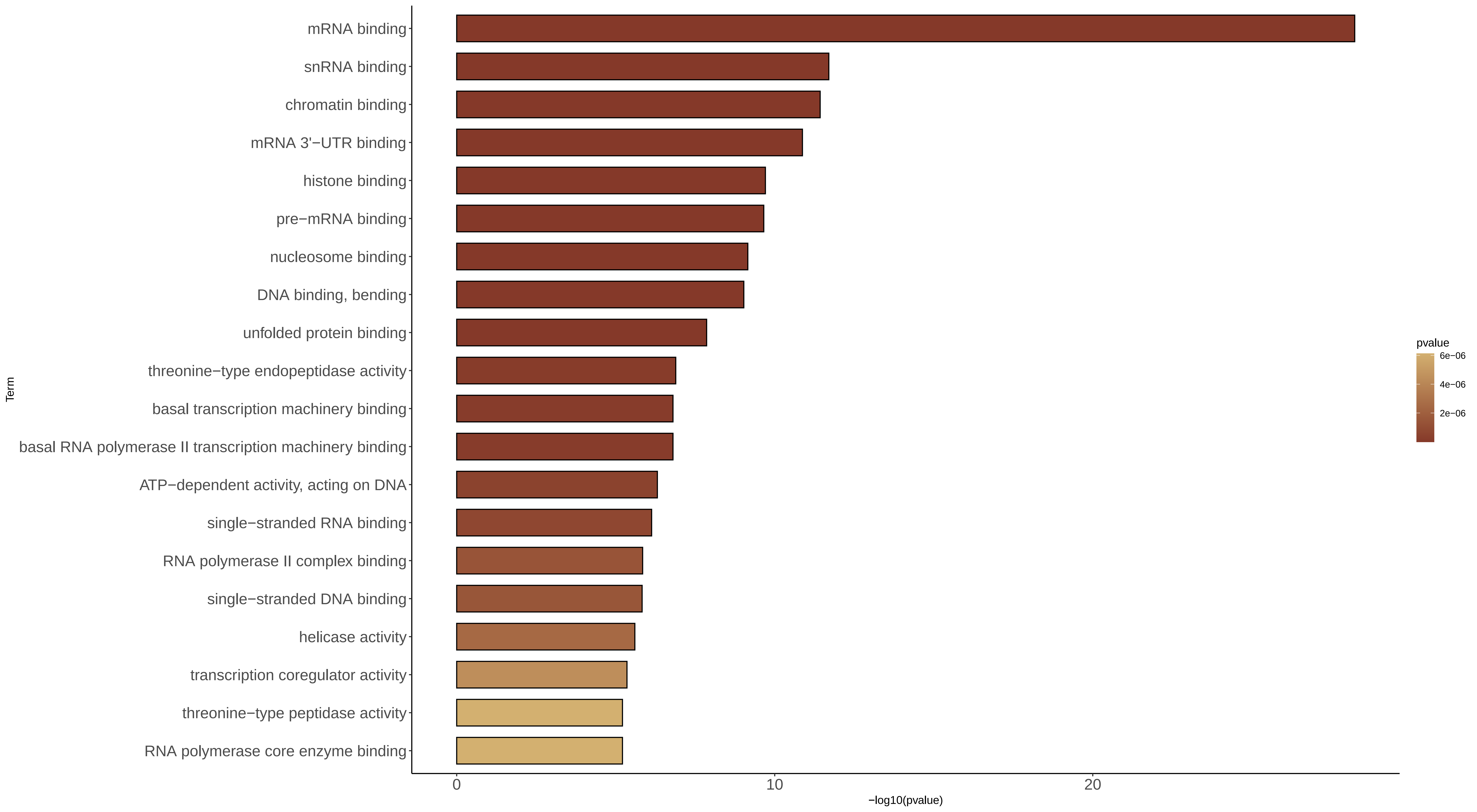
mRNA binding
snRNA binding
chromatin binding
mRNA 3'−UTR binding
histone binding
pre−mRNA binding
nucleosome binding
DNA binding, bending
unfolded protein binding
threonine−type endopeptidase activity
basal transcription machinery binding
basal RNA polymerase II transcription machinery binding
ATP−dependent activity, acting on DNA
single−stranded RNA binding
RNA polymerase II complex binding
single−stranded DNA binding
helicase activity
transcription coregulator activity
threonine−type peptidase activity
RNA polymerase core enzyme binding
Term
0
10
20
−log10(pvalue)
pvalue
6e−06
4e−06
2e−06
